# Mitochondrial-targeted methionine sulfoxide reductase overexpression increases the production of oxidative stress in mitochondria from skeletal muscle

**DOI:** 10.31491/apt.2020.03.012

**Published:** 2020-03-27

**Authors:** Arunabh Bhattacharya, Daniel Pulliam, Yuhong Liu, Adam B. Salmon

**Affiliations:** a The Sam and Ann Barshop Institute for Longevity and Aging Studies, UT Health San Antonio, San Antonio TX, USA.; b Department of Cellular & Structural Anatomy, UT Health San Antonio, San Antonio TX, USA.; c Department of Molecular Medicine, UT Health San Antonio, San Antonio TX, USA.; d Department of Clinically Applied Science Education, University of the Incarnate Word School of Osteopathic Medicine, San Antonio, TX, USA.; e Geriatric Research, Education and Clinical Center, South Texas Veterans Healthcare System, San Antonio TX, USA.

**Keywords:** Superoxide, oxidative stress, mitochondria, protein homeostasis, electron transport chain

## Abstract

**Objective:**

Mitochondrial dysfunction comprises part of the etiology of myriad health issues, particularly those that occur with advancing age. Methionine sulfoxide reductase A (MsrA) is a ubiquitous protein oxidation repair enzyme that specifically and catalytically reduces a specific epimer of oxidized methionine: methionine sulfoxide. In this study, we tested the ways in which mitochondrial bioenergetic functions are affected by increasing MsrA expression in different cellular compartments.

**Methods:**

In this study, we tested the function of isolated mitochondria, including free radical generation, ATP production, and respiration, from the skeletal muscle of two lines of transgenic mice with increased MsrA expression: mitochondria-targeted MsrA overexpression or cytosol-targeted MsrA overexpression.

**Results:**

Surprisingly, in the samples from mice with mitochondrial-targeted MsrA overexpression, we found dramatically increased free radical production though no specific defect in respiration, ATP production, or membrane potential. Among the electron transport chain complexes, we found the activity of complex I was specifically reduced in mitochondrial MsrA transgenic mice. In mice with cytosolic-targeted MsrA overexpression, we found no significant alteration made to any of these parameters of mitochondrial energetics.

**Conclusions:**

There is also a growing amount of evidence that MsrA is a functional requirement for sustaining optimal mitochondrial respiration and free radical generation. MsrA is also known to play a partial role in maintaining normal protein homeostasis by specifically repairing oxidized proteins. Our studies highlight a potential novel role for MsrA in regulating the activity of mitochondrial function through its interaction with the mitochondrial proteome.

## Introduction

Mitochondrial dysfunction is a causative factor of numerous diseases and pathologies and may be a potential primary driver of the aging process itself. One cause of mitochondrial dysfunction in vivo is the loss of mitochondrial protein homeostasis (mito-proteostasis) which is the tightly regulated balance of protein translation, protein quality control, and protein degradation. Mito-proteostasis is complicated by two significant factors: First, > 90% of the mitochondrial proteome is translated in the cytosol and imported to the mitochondria in an unfolded state, in which it is highly susceptible to oxidation [1]. Second, relative to the cytosolic proteome, the mitochondrial proteome is highly enriched in methionine, which is one of the most readily oxidized amino acids due to its side-chain sulfur atom [2, 3]. The sequence of some of the proteins that make up the electron transport chain complexes are compromised of 8–13% methionine of their amino acid content in contrast to an average usage rate of 2.2–2.8% among all cellular proteins [2]. Thus, there is a conundrum as to why the highly oxidative environment of mitochondria would contain such a high amount of easily oxidized proteins.

Among the eukaryotic antioxidant defenses, methionine sulfoxide reductase A (MsrA) plays a unique role in the oxidative repair, and potentially redox regulation, of proteins in the cells. MsrA has been classically defined as a repair enzyme capable of the catalytic reduction of oxidized methionine or methionine sulfoxides [4]. The subsequent discovery of other methionine sulfoxide reductases also pointed out that there is stereo-specificity among such enzymes with MsrA capable of the reduction of primarily the S-epimer of methionine sulfoxide. However, there is a growing amount of evidence that MsrA plays a larger role in the regulation of cellular homeostasis. For example, MsrA has also been shown to have a stereo-specific oxidase activity targeted toward methionine [5]. MsrA may then be capable of regulating protein function through. the redox regulation of methionine residues [6]. In addition, MsrA has been shown to play a protein chaperone-like role in folding proteins; MsrA preferentially repairs oxidized methionine in unfolded proteins and protects these proteins from oxidative protein misfolding [7]. Lastly, MsrA may assist in targeting excessively damaged proteins for proteasome-mediated degradation through ubiquitin-like protein modifications that are distinct from its catalytic modification function [8].

MsrA is expressed ubiquitously in mammals and, at the sub-cellular level, is located natively in both the cytosol and mitochondria. In yeast, the deletion of MsrA significantly increased the production of reactive oxygen species (ROS) and reduced mitochondrial efficiency when yeast are grown on substrates of the electron transport chain (ETC) [9]. These defects were not ascribed to reduced mitochondrial number but rather a reduced number of competent mitochondria in MsrA-deleted yeast. In mammalian retinal pigment epithelial (RPE) cells, the knockdown of MsrA reduced mitochondrial ATP content and the activity of ETC complex IV [10]. Conversely, the adenoviral overexpression of MsrA in RPE cells increased mitochondrial ATP and boosted ETC complex IV activity. Mitochondria isolated from a mouse model of Alzheimer’s disease also lacking MsrA similarly showed reduced oxygen consumption and ETC complex IV activity [11]. Mice lacking MsrA have also shown increased mitochondrial fragmentation and damage following exposure to the DNA damaging agent cisplatin [12]. Collectively, these findings suggest the role of MsrA and, indirectly, the regulation of methionine oxidation, in preserving normal mitochondrial energetic function.

In this study, we used two murine models showing the increased expression of MsrA targeted primarily to either the mitochondria or cytosol. Endogenously, the sub-cellular localization of MsrA is determined by the alternative translation initiation sites, which include (or do not) an N-terminal mitochondrial targeting sequence on the native translated protein. While the native distribution of MsrA is ~3:1 cytosolic to mitochondrial, here, we used two different transgenic MsrA mouse strains to test whether increasing MsrA in either subcellular compartment would alter the mitochondrial bioenergetics or function. We (and others) have reported that TgCyto MsrA mice have increased levels of cytosolic MsrA due to a deletion of the endogenous mitochondrial targeting sequence of MsrA in the overexpressed transgene [13–15]. Conversely, TgMito MsrA exhibits the preferential overexpression of mitochondrial-targeted MsrA due to its preferential expression of the endogenous mitochondrial targeting sequence of MsrA in the transgene [13, 14, 16]. In this study, we tested the energetic and oxidative stress characteristics of mitochondria isolated from the skeletal muscle of these mice to address the potential role of MsrA in murine mitochondrial function.

## Methods

### Animals

All studies in this research were reviewed and approved by the UT Health San Antonio (UTHSA) Institutional Animal Care and Use Committee (IACUC), which is responsible for regularly monitoring housing and animal conditions to ensure all guidelines are met for the safety and health of the animals. All experiments were conducted in compliance with the US Public Health Service’s Policy on Humane Care and Use of Laboratory Animals and the Guide for the Care and Use of Laboratory Animals. We have previously reported the generation and breeding of TgCyto MsrA and TgMito MsrA mice [13, 14, 16]. In this study, young mice (5–7 months of age) were used and fed a normal animal chow (NIA-31) diet for their life. The animals were sacrificed with CO_2_, and muscle and other tissues were collected.

### Mitochondrial function assays

Mitochondria were isolated from freshly collected hind-limb skeletal muscle (gastrocnemius, tibialis, and soleus) using the methods previously described [17]. Briefly, the muscles were homogenized with protease, and the mitochondria were purified through differential centrifugation. H_2_O_2_ release from the mitochondria under specified conditions was assessed using the Amplex Red method as is described in [18]. Mitochondrial substrates were added at the following concentrations: glutamate (2.5 mM), malate (2.5 mM), succinate (5 mM), rotenone (0.5 μM), and antimycin A (0.5 μM). The same concentrations were used for ATP production, membrane potential, and mitochondrial respiration. Superoxide release was measured using electron paramagnetic resonance (EPR) with the use of spin trap 5-diisopropoxyphosphoryl-5-methyl-1-pyrroline-N-oxide (DIPPMPO), as previously described [17]. EPR data was expressed as relative intensity per 20 μg mitochondrial protein and then normalized to values generated from the control mice. ATP synthesis was measured using the luciferin/luciferase assay from Roche according to the manufacturers’ instructions. The slope of the kinetic curve generated was converted to ATP measurements using the standards provided in the kit. Membrane potential was measured by the fluorescence of the quench-dye Safarin O, as previously described [18]. The respiratory control ratio (RCR) was measured as the ratio of the mitochondrial State 3/State 4 respiration rates measured by the Clark electrode, as described previously [17, 18]. Briefly, State 3 respiration was measured in the presence of 0.3 mM ADP, and State 4 respiration was measured as oxygen consumption following the expenditure of ADP. Aconitase catalyzes the reversible isomerization of citrate into isocitrate. In most tissues, aconitase is usually present in both the mitochondrial matrix and the cytoplasm. However, in skeletal muscle, only mitochondrial matrix aconitase is present. Aconitase activity was assayed (in Triton-X-100-treated samples) by measuring NADP+ reduction via citrate in the presence of isocitrate dehydrogenase using a fluorometric method (excitation at 355 nm and emission at 460 nm). Skeletal muscle homogenates (~1.0 mg of protein/ml) were aliquoted in 96-well plates (100 μl of pH 7.44, 125 mm KCl, 10 mm HEPES, 5 mm MgCl2, 2 mm K2HPO4) and incubated at 30° C for up to 40 min. After incubation, aconitase activity measurements began via the addition of 1 volume (100 μl) of 50 mm Tris, 0.6 mm MnCl2, 60 mm citrate, 0.2% Triton X-100, 100 μm NADP+, and 1 unit of isocitrate dehydrogenase (Sigma). Fluorometric measurements were then initiated immediately (Fluoroskan-FL Ascent type 374 microplate reader). As a negative control, we used a blank consisting of the same buffer minus the isocitrate dehydrogenase. Assessment of the slope of NADPH fluorescence change was used for the assessment of aconitase activity.

### Mitochondrial complex assays

The activity of the ETC complexes was measured as previously described [17]. In brief, total mitochondrial proteins were assessed for complex I activity by monitoring the oxidation of nicotinomide adenine dinucleotide (NADH), with ubiquinone-2 as the electron acceptor in the presence of diclorophenolindophenol (DCIP). Complex II activity was assessed by measuring the succinate-dependent reduction of DCIP, using ubiquinone-2 as the electron receptor. Complex III activity was measured by the reduction of cytochrome c^3+^ at 550 nm, using D-ubiquinol-2 as an electron acceptor. Complex IV was measured by monitoring the oxidation of cytochrome c^2+^. All assays were measured via spectrophotometry, and they are described in greater detail as in [17]. The final rates for all activities were normalized to the average values obtained for the wild type (control) animals.

### Statistical analysis

All data was analyzed by a one-way ANOVA or Student’s t-test, as appropriate. Statistical significance was given to data where p < 0.05. Post-hoc analysis of the ANOVA was performed using the method of Holm-Sidak.

## Results

**Figure 1.**
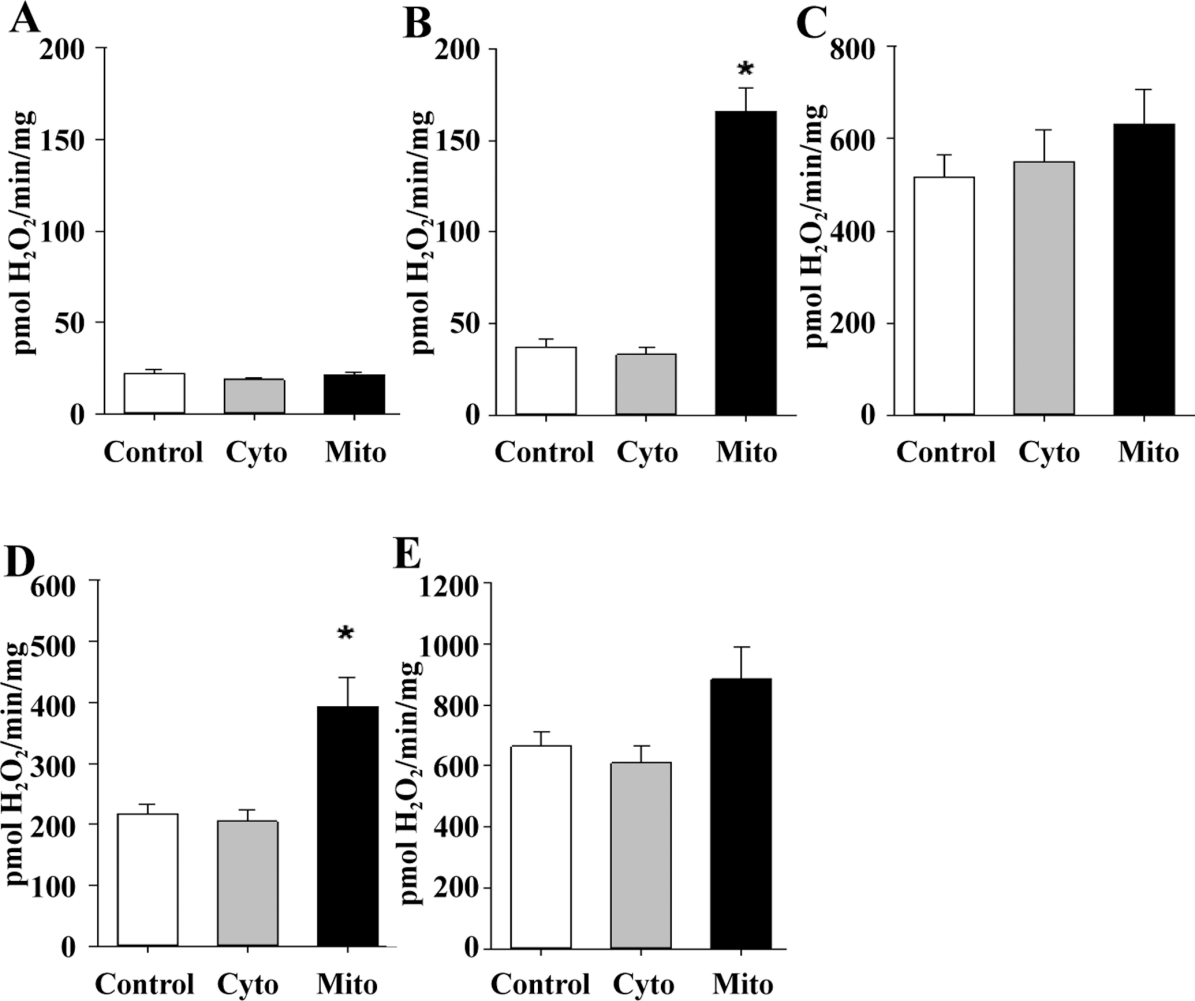
Mitochondrial production of H_2_O_2_ with no substrates provided (**A**; state 1) or provided with glutamate and malate (**B**; Glu/Mal), glutamate, malate and rotenone (**C**; Glu/Mal/Rot), succinate and rotenone (**D**; Suc/Rot) or succinate, rotenone and antimycin A (**E**; Suc/Rot/AA). Skeletal muscle mitochondria was isolated from wild type (Control),TgMito MsrA (Mito) and TgCyto MsrA (Cyto) mice. Bars represent average values for n = 5 for each group ± SEM. Asterisks indicate group differs significantly from others by ANOVA (p < 0.05).

Based on reports suggesting a lack of MsrA causes mitochondrial dysfunction, we tested whether isolated mitochondria from mice with elevated MsrA levels, either primarily in the cytosol or primarily in the mitochondria, would differ from those of control mice. We first addressed the rates of H_2_O_2_ production as a marker of mitochondrial-derived reactive oxygen species (ROS) production. Under basal respiration, H_2_O_2_ production was low and did not differ among the genotypes (Figure 1A). When provided with glutamate and malate (substrates for ETC complex I), H_2_O_2_ production was significantly elevated; interestingly, under these conditions, H_2_O_2_ production was nearly four times greater in TgMito MsrA mitochondria than in the control or TgCyto MsrA mitochondria (Figure 1B), we found that mitochondria from all three genotypes showed similarly high rates of H_2_O_2_ production when glutamate, malate and rotenone, an inhibitor of ETC complex I, were added (Figure 1C). H_2_O_2_ production was still significantly elevated in TgMito MsrA compared to control and TgCyto MsrA mitochondria when succinate, a substrate of the ETC complex II, was added (Figure 1D). With the addition of antimycin A, an inhibitor of ETC complex III, we found a similar difference between TgMito MsrA and control ROS production; however, this did not reach statistical significance (Figure 1E). For all assays, we found no difference in ROS production between the mitochondria of the TgCyto MsrA and control mice. Because mitochondria do not produce H_2_O_2_ directly, we also measured superoxide production from isolated mitochondria using electron paramagnetic resonance (EPR).

**Figure 2.**
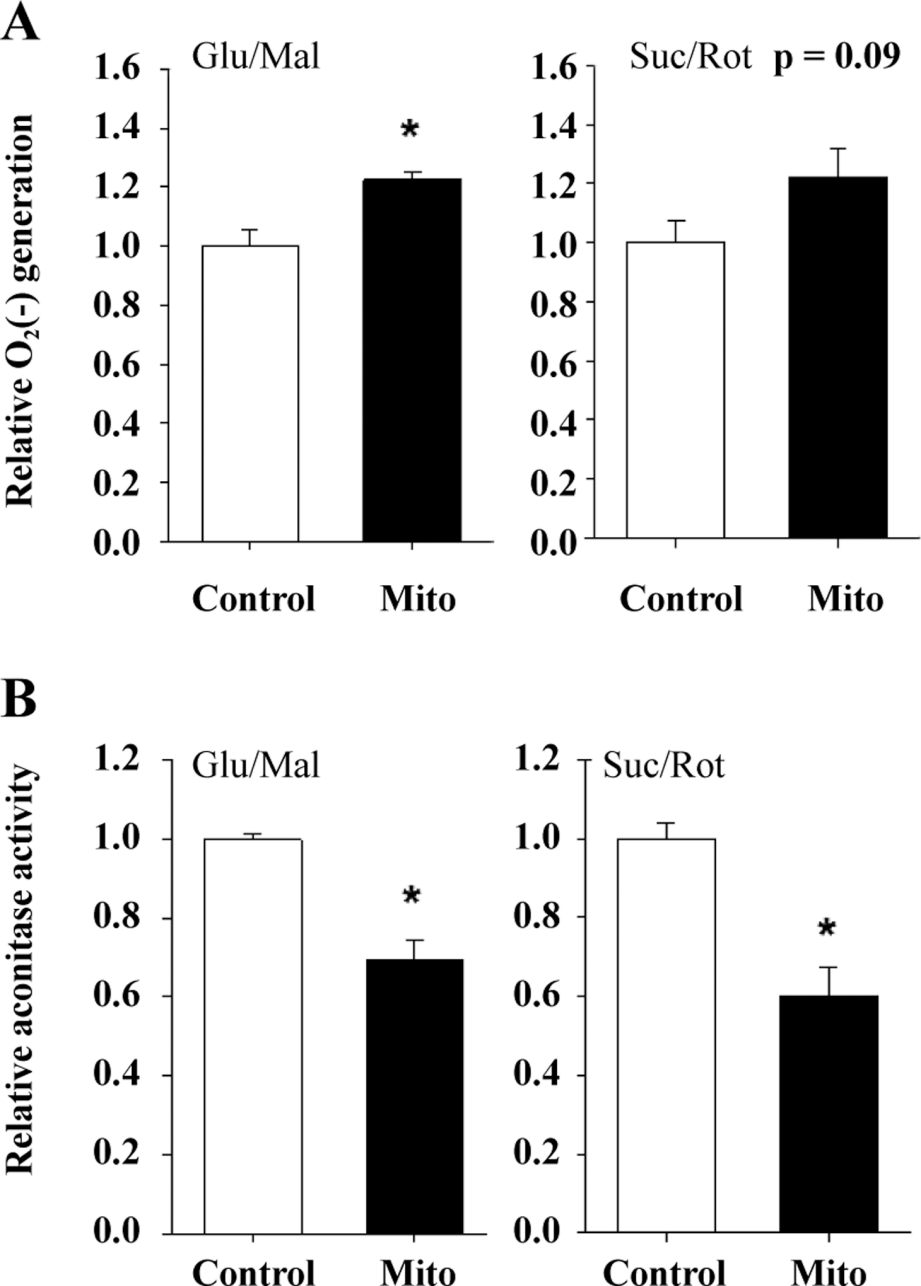
**(A)** Superoxide generation from mitochondria isolated from muscle of wild type (Control) and TgMito MsrA (mito) mice provided glutamate and malate (Glu/Mal) or succinate and rotenone (Suc/Rot). **(B)** Aconitase activity in isolated mitochondria provided glutamate and malate (Glu/Mal) or succinate and rotenone (Suc/Rot). Bars represent average values for n = 6 for each group ± SEM. Asterisks indicate significant difference as measured by t-test (p < 0.05).

Whether substrates of ETC complex I (glutamate and malate) or complex II (succinate with rotenone to inhibit complex I) were provided, superoxide generation was higher in the mitochondria of TgMito MsrA mice than the control using this method (Figure 2A). We also measured whether superoxide are released into the mitochondria through a measurement of aconitase activity Aconitase activity is inhibited in the mitochondrial matrix via interaction with superoxide [19]. The significantly reduced activity of aconitase in TgMito MsrA mitochondria again suggested elevated levels of superoxide with increasedmitochondrial MsrA (Figure 2B).

**Figure 3.**
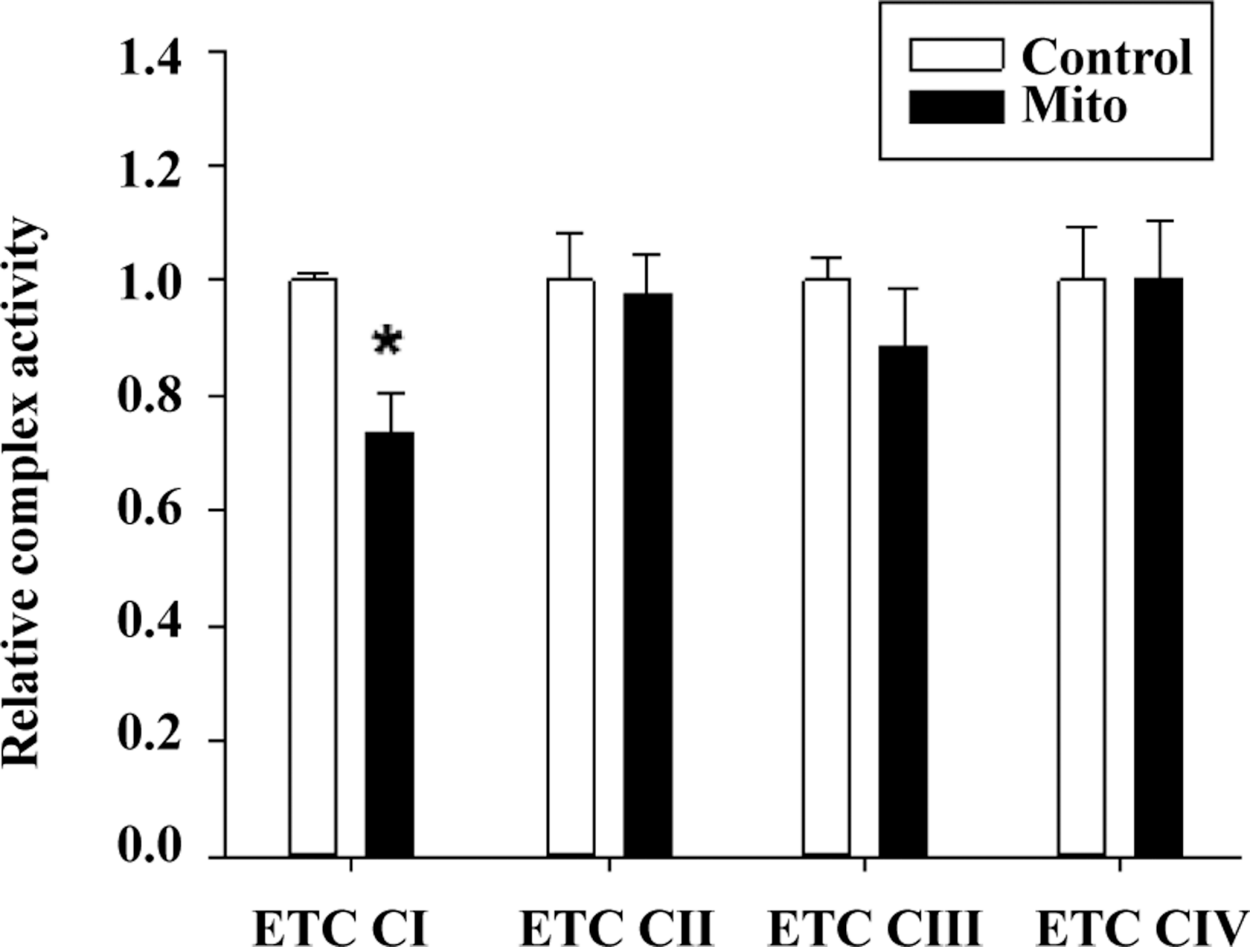
Electron transport chain complex activity in isolated mitochondria from wild type (Control) and TgMito MsrA mice (Mito). Bars represent average values for given complex for n=5 for each group ± SEM. Asterisks indicate significant difference as measured by t-test (p < 0.05).

Of the potential sources of mitochondrial superoxide production, we focused on the activity of the ETC complexes, especially ETC complex I and III, as potential mechanisms for these differences. In isolated mitochondria, we found a significant reduction in ETC Complex I activity in TgMito MsrA mitochondria compared to the controls (Figure 3). ETC Complex II, III, and IV were all similar between the two genotypes, suggesting this could be the potential source of increased superoxide generation in TgMito MsrA mitochondria.

**Figure 4.**
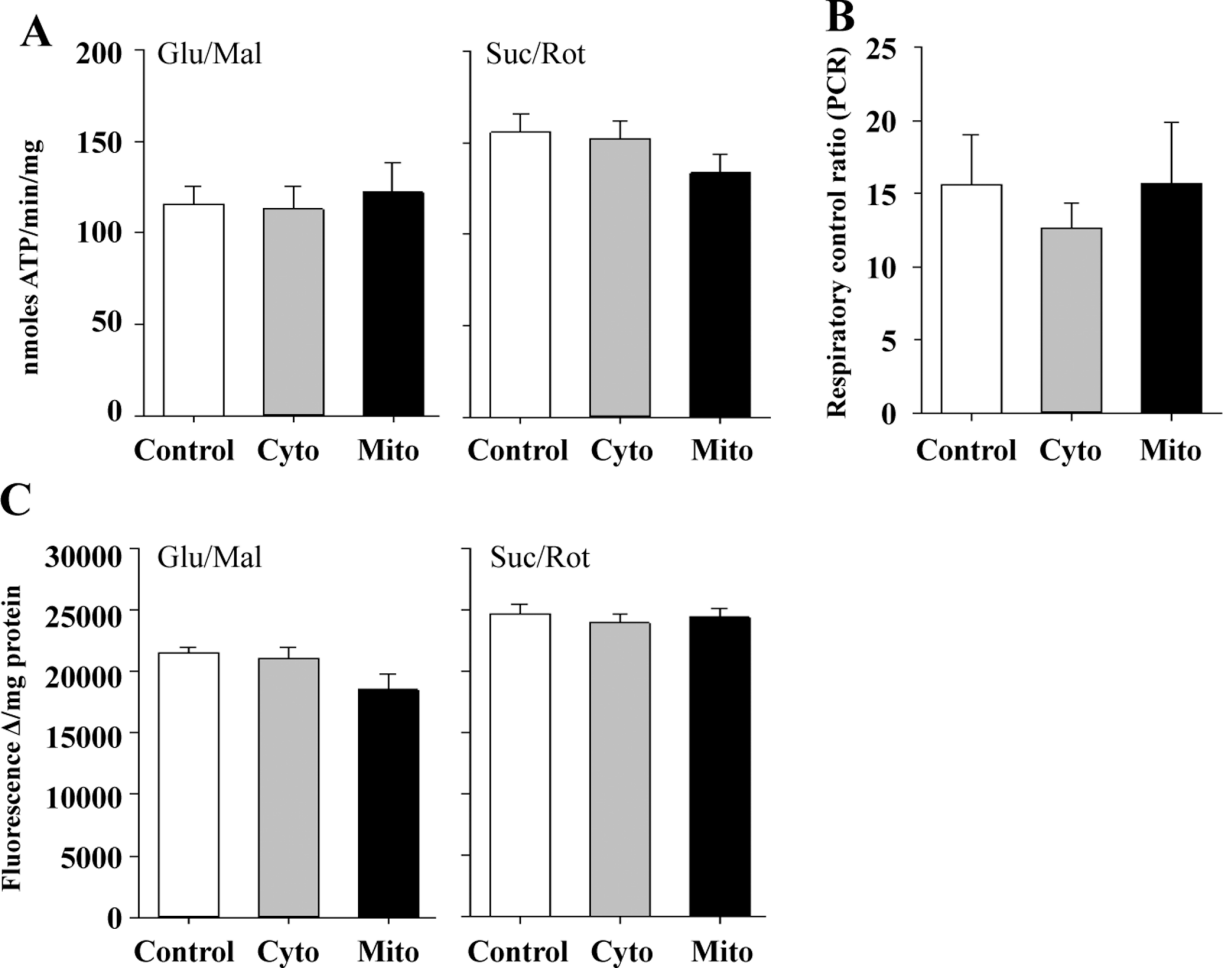
**(A)** ATP production from isolated mitochondria provided glutamate and malate (Glu/Mal) or succinate and rotenone (Suc/Rot). **(B)** Respiratory control ratio (RCR) of mitochondria provided glutamate and malate. **(C)** Membrane potential of isolated mitochondria provided glutamate and malate (Glu/Mal) or succinate and rotenone (Suc/Rot). Skeletal muscle mitochondria was isolated from wild type (Control),TgMito MsrA (Mito) and TgCyto MsrA (Cyto) mice. Bars represent average values for n = 5 for each group ± SEM. Asterisks indicate group differs significantly from others by ANOVA (p < 0.05).

Despite the increase in free radical production and the reduction in ETC Complex I activity, we found little detrimental effect on the actual bioenergetics of mitochondria from TgMito MsrA mice. Moreover, ATP production did not differ among the three genotypes of mice when generating ATP from ETC Complex I (glutamate and malate) or complex II (succinate and rotenone to inhibit Complex I). Similarly, increasing MsrA levels had no effect on the respiratory control ratio (RCR) of mitochondria or the mitochondrial membrane potential (Figure 4).

## Discussion

Contrary to our initial prediction, our findings suggest that elevated levels of MsrA in the mitochondria lead to the increased generation of mitochondrial-derived free radicals without significantly affecting mitochondrial bioenergetics. These outcomes raise interesting questions, the first of which asks why increasing mitochondrial MsrA might reduce ETC complex I activity. Of note, mammalian complex I is highly enriched in overall methionine content of its proteins, with up to four times the quantity of methionine compared to the methionine content of the total cellular proteome [2]. It has been proposed that a high methionine content might act as an antioxidant or “free radical sink” within the highly oxidative environment of mitochondria [2, 20]. Moreover, the majority of proteins making up this complex are imported to the mitochondria as unfolded proteins [21]. As MsrA has been shown to preferentially bind unfolded proteins [7], it may be that the overabundance of MsrA in mitochondria from TgMito MsrA mice may be physically bound to these components, potentially inhibiting the proper folding of the complex structures. While the levels of MsrA expressed in these mice may be much higher than normally expressed in vivo [14, 16], these results raise the intriguing possibility that MsrA plays a protein chaperone-like function in the assembly of mitochondrial protein complexes.

We have previously shown that TgMito MsrAs are protected against glucose metabolic dysfunction caused by either high fat diets or advanced age [14, 16]. In light of the results presented here, these physiological outcomes are intriguing because there has been some consensus that oxidative stress is associated with, and may cause, metabolic dysfunction, including insulin resistance [15, 22]. However, there is also a growing amount of evidence that H_2_O_2_ signaling is required for many normal cellular functions, including metabolic function [23, 24]. Because our assays were performed using isolated mitochondria, it is still possible that these findings are an artifact of the experimental procedure. Thus, it would be of interest to determine in vivo free radical production in living TgMito MsrA mice to better define this potential relationship.

More broadly, our data aligns with an expanding number of findings that seem to refute the oxidative stress theory of aging, at least in murine models. While this theory originally received much attention, in part due to its simplicity, support for it has been largely equivocal. Most aging studies conducted in mice with genetically altered enzymatic antioxidants have shown no consistent effect on longevity [25]. Even in transgenic mice with mitochondrial-targeted antioxidant overexpression, there has been little consensus as to their effect on lifespan [14, 26, 27]. On the other hand, aging is a biological process, and there is clear interest in understanding how age-related changes in health (and not just lifespan) might be regulated by processes such as mitochondrial oxidative stress [28, 29]. In this regard, there is evidence that increased oxidative stress drives multiple health deficits in mouse models of aging [30, 31]. We have reported a potential aging benefit of TgMito MsrA mice on metabolic function, while others have shown these mice to not be protected from a cardiac ischemia-reperfusion model [13, 14]. It would be of interest to take a more holistic approach toward functional aging assessments to determine the role of mitochondrial MsrA in the regulation of theaging process.

The actions of MsrA in the mitochondria may be beyond that of the catalytic reduction of methionine sulfoxide. There is a developing set of evidence that methionine oxidation can regulate protein function with stereo-specific oxidation and reduction using methionine sulfoxide reductases as a key regulator [5, 6, 32]. In addition, an increasing amount of clues that MsrA directs protein degradation through ubiquitin-like modifications suggest that MsrA plays a far more central role in proteostasis than previously believed [8]. Moreover, the importance of methionine metabolism in regulating cellular homeostasis, for example transsulfuration and hydrogen sulfide generation, suggests methionine sulfoxide reductases could function in a previously unrecognized pivotal regulatory mode for maintaining normal cellular function and communication [33, 34].
